# Microstructure and Hardness of Nickel-Based Coatings Prepared by Laser Additive Manufacturing on Water-Cooled Substrate: An Experimental and Numerical Study

**DOI:** 10.3390/ma17235692

**Published:** 2024-11-21

**Authors:** Mingjun Yan, Ruifeng Li, Jiajunqi Guo, Bin Liu, Xiaoqiang Zhang, Yue Zhao, Taotao Li, Lei Qiao, Seyed Reza Elmi Hosseini

**Affiliations:** 1 School of Materials Science and Engineering, Jiangsu University of Science and Technology, Zhenjiang 212100, China; 221110601128@stu.just.edu.cn (M.Y.); 231210601408@stu.just.edu.cn (J.G.); liubindely@163.com (B.L.); 202100000022@just.edu.cn (X.Z.); zhaoyue90214@just.edu.cn (Y.Z.); litaotao@just.edu.cn (T.L.); 202200000208@just.edu.cn (L.Q.); 2 School of Metallurgy and Materials Engineering, Iran University of Science and Technology, Narmak, Tehran 13114-16846, Iran; elmihosseini@iust.ac.ir

**Keywords:** laser additive manufacturing, Ni45 alloy powder, water-cooled substrate, temperature field

## Abstract

The excess heat generated during the laser additive manufacturing process is prone to cause coating defects; a water-cooled substrate can effectively remove the excess heat and improve the hardness of the coating. In this study, the effects of water-cooled substrate on the microstructure and hardness of laser additive manufactured nickel-based coatings were investigated by experimental and numerical simulations. The results showed that the water-cooled substrate decreased the size of columnar crystals and increased the number as well as the length of secondary dendrite crystals at the bottom of the nickel-based coatings. There was also a noticeable increase in the size of equiaxed grains and the quantity of the solid solution in the middle of the coatings. The hardness value of the coating increased at the water velocity of 200 mL/s and 500 mL/s and finally decreased at 700 mL/s. A finite element model was established by ABAQUS software to numerically simulate the temperature field of the laser additive manufactured nickel-based coating with the water-cooled substrate. The results revealed significant differences in the temperature distribution of the coatings with different velocities. As the water velocity increased, the peak temperature at the center of the coating’s molten pool gradually decreased. In addition, the cooling rate of the specimens increased with the application of the water cooling, leading to a more concentrated temperature distribution near the laser heat source.

## 1. Introduction

Nickel-based alloys are recognized as alloys with more than 30 wt% nickel content, which have good tensile properties, fatigue resistance, and certain oxidation resistance and corrosion resistance at 650–1000 °C [1,2,3]. Common nickel-based alloys have excellent high-temperature resistance and corrosion resistance. Therefore, these are widely used in high-temperature aerospace engines, glycerol steam reforming and gas turbines, oil extraction equipment in marine corrosive environments, and wear-resistant equipment in the petrochemical and chemical process industries [4,5,6]. Also, nickel-based coatings protect substrate alloys from fatigue cracking by enhancing surface hardness, corrosion resistance, and wear resistance. These coatings create a hard, smooth surface that reduces friction, delays crack initiation, and prevents surface deformations under cyclic loading. Nickel coatings also act as thermal barriers, shielding substrates from thermal fatigue in high-temperature environments. Additionally, they help in redistributing stress across the material surface, minimizing localized stress concentrations that can lead to fatigue cracks [7,8]. As an advanced metal additive manufacturing technology, laser additive manufacturing has the advantages of strong metallurgical bonding ability, easy large-area repairing, low dilution rate, and good thickness controllability compared with traditional surface technologies such as electroplating, thermal spraying, physical vapor deposition, and arc welding, which can improve the surface properties of materials [9,10,11,12]. As a common high-quality carbon structure-bearing steel, the microhardness of 45 medium carbon steel is about 200 HV_0.2_, R_m_ ≥ 600 MPa, R_p0.2_ ≥ 355 MPa, which has excellent mechanical properties. At the same time, this type of steel has good cold formability and low price and is widely used in various fields [13,14,15]. Ni45 self-melting alloy has a strong combination of strength and toughness and has a high physical and chemical compatibility with 45 medium carbon steel. Therefore, it is easy to obtain a crack-free Ni45 alloy coating with high density and good metallurgical bonding [16,17].

The cooling rate plays an important role in the process of alloy melting and recrystallization, affecting the recrystallization microstructure. Increasing the cooling rate can refine the crystal grain, thereby improving the mechanical properties and thermal stability of the alloy. Wang et al. [18] studied the effect of the cooling rate on the microstructure and mechanical properties of cast Al-Li-Cu-Mg alloy. The results show that the increase in cooling rate refines the grain size and increases the volume fraction of the second phase in the casting. Thus, this improves the tensile properties of the sample. Su et al. [19] investigated the effect of the cooling rate on the microstructure and mechanical properties of M50 steel after the isothermal stage of the vacuum austenite backfired. The results depict that with the increase in cooling rate, the hardness of the austenitic tempered steel increases; however, the hardness of tempered steel decreases. This is related to the formation of martensite and its tempering decomposition. Han et al. [20] fabricated AlCrFe_2_Ni_2_ medium entropy alloy (MEA) using L-PBF at different cooling rates by changing the process parameters. The results infer that the cooling rate has a significant effect on the phase composition, microstructure, and mechanical properties. Qin et al. [21] illustrated the relationship between the microstructure of silver copper eutectic alloy and the secondary cooling rate. The results reflect that the cooling rate not only affects the selection of the primary phase and the eutectic lamellar spacing but also affects the mode of the eutectic nucleation and growth.

Different from traditional directional solidification, the laser additive manufacturing process cannot maintain high-temperature transient, heterogeneous nucleation, and heat accumulation during the forming process of parts. These will have a very serious impact on the repair. By applying the water cooling to the bottom of the substrate, the excess heat attracted by the substrate during the laser additive manufacturing process can be taken away to achieve a real-time cooling effect and improve the temperature gradient [22]. Meanwhile, ABAQUS, ANSYS, COMSOL Multiphysics, and artificial intelligence can be used to predict and simulate the temperature change during the laser additive manufacturing process, which is conducive to further studying the effect of the water cooling on the laser additive manufacturing process [23].

Chen et al. [24] investigated the effect of continuous water flow on substrate cooling during the IN718 laser additive manufacturing process. Analyses via OM, SEM, and EBSD showed heightened crystal orientation, forming ordered dendrites and monocrystalline texture. Solidification cracking minimally impacted properties, primarily at the apex, while liquation cracking affected the HAZ, requiring meticulous control. Liquation cracking susceptibility correlated with grain boundary misorientation and localized stress concentrations during solidification. Wu et al. [25] prepared Al_2_O_3_-YAG eutectic ceramics by laser-directed energy deposition on substrates with water cooling. The test results showed that the microhardness of Al_2_O_3_-YAG increased by about 10.6%, from 19.44  ±  1.63 GPa to 21.50 ± 2.70 GPa. Nie et al. [26] applied the water cooling across the single crystal Rene N5 substrate during laser-directed energy deposition of Ni-based coatings. Single-layer remelting, one-layer deposition, two-layer deposition, and eight-layer deposition were investigated to explore the mechanism of grain growth. The solidification conditions, including the temperature field, temperature gradient, and the solidification rate of the DED process, were numerically analyzed by a finite element model. The results exhibit that the proportion of columnar crystal zone increases significantly after the application of the water cooling of the substrate. The numerical simulation results expose that the temperature gradient in the [001] direction increases significantly after forced water cooling. Qing et al. [27] studied the ultrasonic vibration-assisted laser-directed energy deposition technology. The results depict that the relative error of the model when calculating the profile of the coatings is less than 10%, which can accurately calculate the temperature field of the coatings. The average microhardness of the cladding track increases from 508.3 HV_0.2_ at 0 W ultrasonic power to 547.9 HV_0.2_ at 2600 W. Chen et al. [28] established a DED model combining finite element (FE) and thermo-mechanical–metallurgical (TMM) analysis and verified it by thermal imaging system and X-ray diffraction (XRD) stress measurement. The combination of experiment and prediction simulation reveals nonlinear thermo-metallographic evolution and the formation mechanism of the compressive tensile residual stress of low-temperature phase change alloy during laser-directed energy deposition. This also reveals the great potential of low-temperature phase change alloy powder to reduce the tensile residual stress of the laser-directed energy deposition parts through austenite to martensite transformation during the cooling.

In this study, the substrate was cooled by water during the laser additive manufacturing process, and the effects of different cooling velocities on the microstructure and microhardness of the coating were analyzed. The temperature field distribution was simulated by ABAQUS, which provided data support and new ideas for improving the properties of Ni45 coating.

## 2. Methods

### 2.1. Experimental Materials

**Figure 1:**
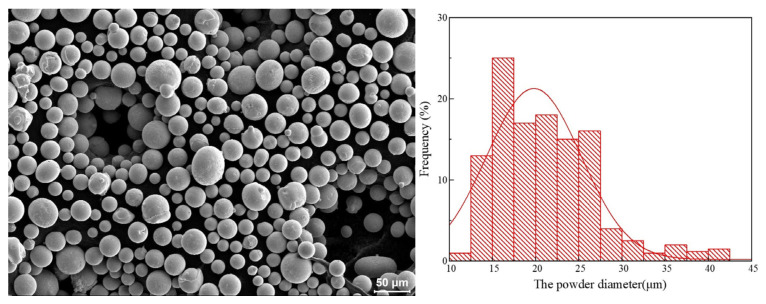
SEM morphology and diameter distribution of Ni45 alloy powder.

The powder used in this study is Ni45 alloy powder, and its SEM image is depicted in Figure 1 (left). As shown in Figure 1, the powder shape is spherical or ellipsoidal. This shape of the powder ensures the fluidity and continuity of the powder feeding during the laser additive manufacturing process and also makes the powder melt more efficiently. The particle analysis software called Image J (1.54, National Institutes of Health, Bethesda, MD, USA) measure was used to measure the powder diameter. The measured data were mapped, as demonstrated in Figure 1. The particle size distribution of the alloy powder is between 15 μm and 30 μm, and the average size is 24.61 μm. The substrate in this experiment is 45 medium carbon steel (AISI 1045) of 140 × 140 × 10 mm.

The chemical composition of Ni45 is illustrated in Table 1.

### 2.2. Laser Additive Manufacturing Process on Water-Cooled Substrate

**Figure 2:**
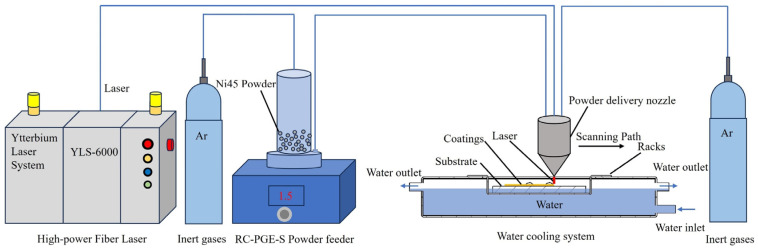
A laser additive manufacturing system based on the water-cooled substrate.

Figure 2 reveals the experimental platform of the laser additive manufacturing system based on a water-cooled substrate, including a water tank, a laser head and its source, the powder feeding system, and the steel substrate. The laser and powder feeding system includes a high-power optical fiber laser YSL-6000 (IPG, Marlborough, MA, USA), an IRB2600 (ABB, Zurich, Switzerland) six-axis robot, and a coaxial focusing powder feeding system RC-PGF-S (Nanjing Zhongke Yu Chen Laser Technology, Nanjing, China). The Ni45 powder is sent to the coaxial powder feeding head preset on the ABB robot for laser additive manufacturing through the coaxial focusing powder feeding system.

**Figure 3:**
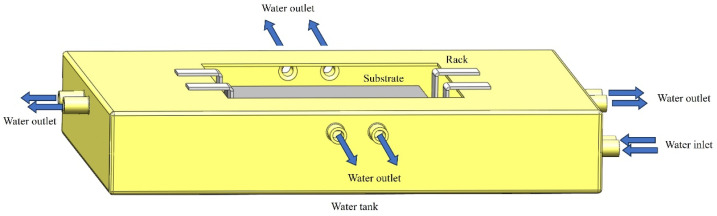
A schematic diagram of the water cooling system.

The design of the water cooling system is displayed in Figure 3.

The size of the water cooling system is 350 × 350 × 100 mm, which is composed of a water tank, two racks, two water inlets, and eight outlets. The water tank is welded with 2 mm thick brass. The thin water tank and good thermal conductivity of the brass ensure that the cooling water in the water tank and the outside air is well exchanged during the laser additive manufacturing process. The rack is made of a 2 mm thick 316L stainless steel plate by hot working, which has excellent toughness. It ensures that the substrate undergoes only a relatively small degree of deformation during the laser additive manufacturing process. The substrate was placed on the rack, then the high-pressure water valve opened. Afterward, the cooling water passed from the water valve into the water pipe. The water flow is divided into two streams through the three-way connector and then enters the water tank at the same time to ensure that the water flow entering the water tank is stable. The cooling water in the water tank contacts with the bottom of the substrate after reaching a certain liquid level height. When the liquid level height is higher than the outlet, the cooling water flows out through 8 outlets to obtain a relatively stable liquid level height. Therefore, the lower part of the substrate is subjected to direct contact with the cooling water.

**Figure 4:**
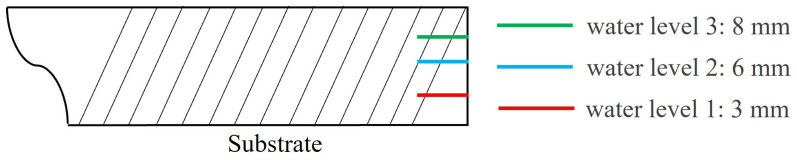
A schematic diagram of the water level.

Three different water levels were designed in this study. In the preparation stage of the experiment, the water level lines were engraved on the edge of the three substrates with a knife and a scale. The heights of the three water levels from the substrate were 3 mm, 6 mm, and 8 mm. The relative height of the water level is shown in Figure 4. Employing a volumetric cylinder and a timer, the water output of a single water outlet per unit of time was measured and repeated 10 times, and the average value was finally calculated for precision. Subsequently, the total water velocity was determined for three water levels, 200 mL/s, 500 mL/s, and 700 mL/s. The liquid level height was changed by controlling the water valve. The liquid level coincides with the scale line, and then the liquid level height reflects the velocity of the cooling water.

In this study, different process parameters were designed to compare the characteristics of the normal laser additive manufacturing process and the laser additive manufacturing process on the water-cooled substrate. The laser additive manufacturing process parameters are depicted in Table 2.

The laser spot size used in this investigation was 5 mm × 5 mm, and the distance of the powder feeding head moving during the experiment was set as 60 mm. The area of the substrate is much larger than the area of the coating to ensure that the heat transfer at the bottom of the coating is almost uniform during the laser additive manufacturing process.

### 2.3. Finite Element Analysis

#### 2.3.1. Finite Element Model

**Figure 5:**
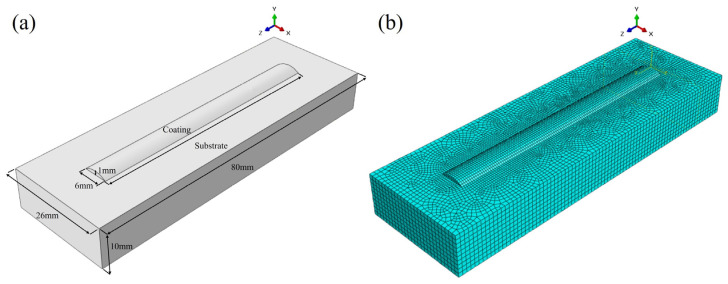
Schematic diagram of the numerical model for laser additive manufacturing: (**a**) geometric model and (**b**) finite element analysis meshing.

It is well known that for the same material, solidification conditions play a leading role in influencing the microstructure and properties. In order to better analyze the effect of the water cooling on the solidification behavior of the laser additive manufactured Ni45 coatings. ABAQUS (2020, ABAQUS, Paris, France)was used in this study to simulate the thermal cycling process of the solidification behavior. The finite element model was established according to the height and width of the coating. The finite element model is shown in Figure 5, in which the substrate size is 80 mm × 26 mm × 10 mm, and the coating has a height of 1 mm, a width of 6 mm, and a length of 60 mm.

ABAQUS software was used to simulate the temperature, residual stress, and deformation during the laser additive manufacturing process. The accuracy of the mesh has an important influence on the simulation. If the mesh size is too large, the accuracy of the simulation will decline, and the calculation time will be greatly increased if the mesh size is too small. Therefore, the meshing has an important influence on the finite element simulation [29].

The current simulation uses temperature–displacement coupling for the finite element analysis. The mesh type used in the middle of the coating is an irregular hexahedron, which is an 8-node thermally coupled hexahedron element with three-way linear displacement and three-way linear temperature. The mesh type used in the substrate is a cuboid. A total of 57,260 meshes were divided in this numerical simulation, including 2640 meshes in the cladding layer and 54,620 meshes in the substrate.

**Figure 6:**
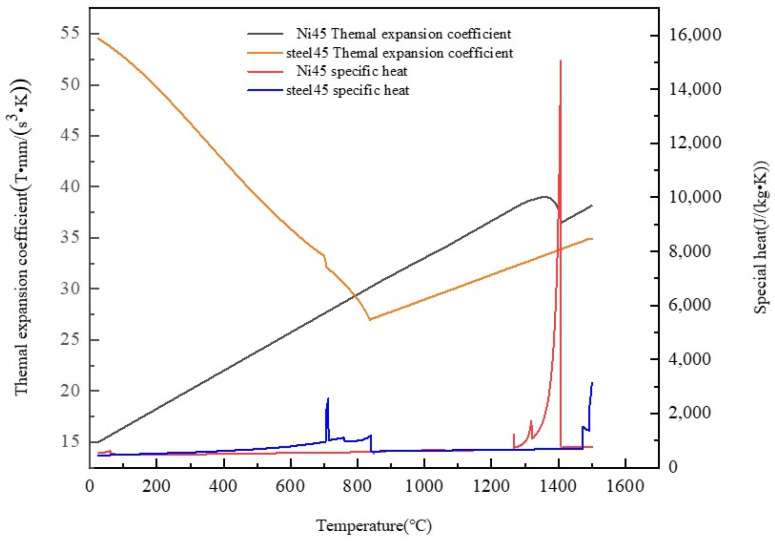
Material properties of Ni45 alloy powder and 45 medium carbon steel.

Through the simulation calculation of 45 medium carbon steel and Ni45 alloy by JMat Pro 7.0, the material properties of the substrate and deposition powder with temperature change are obtained, which are shown in Figure 6.

#### 2.3.2. Heat Source Selection

**Figure 7:**
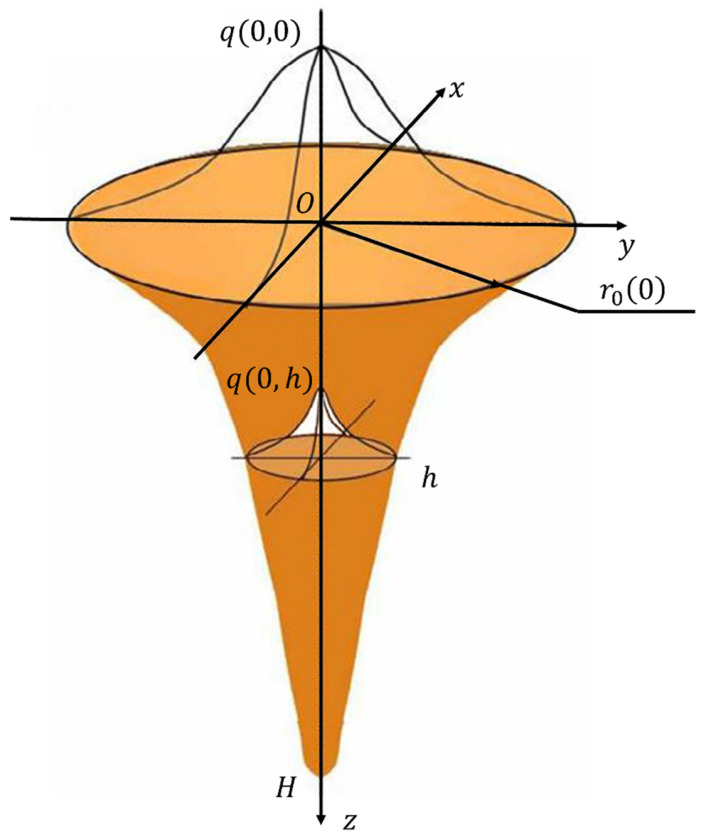
The schematic diagram of Gaussian body heat source.

The heat source model used in this simulation is the Gaussian heat source model. The Gaussian body heat source is a body heat source that rotates the Gaussian curve around its symmetry axis according to the general laser beam energy peak distribution law. It can accurately reflect the morphology of the coating and improve the calculation accuracy of temperature [30]. The Gaussian body heat source energy is distributed inside the rotating Gaussian surface, as depicted in Figure 7.

Assuming that the radius of the heat source on the incident surface of the Gaussian body heat source laser is *r*_0_ and the depth of the heat source is *H*, the heat flow distribution function is as follows [31]:

(1) $$q\left(r,z\right)=\frac{9Q}{\pi r_{0}^{2}H}\cdot \frac{e^{3}-1}{e^{3}}exp\left(-\frac{9r^{2}}{r_{0}^{2}\ln\left(\frac{H}{r}\right)}\right)$$

where $r_{0}$ is the radius of the surface heat source, and H is the depth of the heat source.

The cross-sections of this heat source in the depth direction are concentric circles, the heat flow distribution obeys the Gaussian distribution, and the heat flow density at the center of the circle is the largest.

#### 2.3.3. Boundary Conditions

It can be seen from Section 2.2 that the water velocities of the three water levels are 200 mL/s, 500 mL/s, and 700 mL/s, and the fluid flow rate calculation formula is

(2) $$v=\frac{V_{water}}{s}$$

where *v* is the water velocity, $V_{water}$ is the flow rate, and s is the longitudinal cross-sectional area of the fluid. Combined with Figure 4, the fluid flow rates corresponding to the three water levels are 4.76 m/s, 5.95 m/s, and 6.25 m/s.

#### 2.3.4. Calculation of Convection Parameters

When dealing with any convection problem, the first step is to determine whether the boundary layer is laminar or turbulent. There are obvious differences between laminar and turbulent states, and the heat transfer coefficient is quite different [32]. The transition from laminar to turbulent flow is ultimately due to the triggering mechanism. The occurrence of turbulence depends on the triggering mechanism, which is enhanced or weakened in the flow direction and which depends on the Reynolds number as follows:

(3) $${Re}_{x,c}\equiv \frac{\rho u_{\infty }x_{c}}{\mu }$$

where, for a flat plate, $x_{c}$ is the location at which the transition from laminar to turbulent flow is assumed to begin, $u_{\infty }$ is the free steam value, μ is a fluid property known as the dynamic viscosity, and ρ is the density of the liquid.

The Reynolds number, which represents the ratio of inertial to viscous forces, is a key parameter in determining flow characteristics. When the Reynolds number is small, viscous forces dominate over inertial forces, dissipating disturbances and maintaining a laminar flow state. Conversely, at large Reynolds numbers, inertial forces become significant enough to amplify disturbances, leading to a transition from laminar to turbulent flow. This transition typically begins at a specific location *x_c_*, which is determined by the critical Reynolds number, $R_{e_{x,c}}$. In boundary layer calculations, the critical Reynolds number is commonly taken as 5 × 10^5^.

To analyze this transition and its impact on convection, a method for converting the water flow rate into an average convection coefficient is developed. This process involves three key steps. Firstly, the transition location *x_c_* is determined using the critical Reynolds number. Secondly, the local convection coefficients are calculated separately for the laminar and turbulent regions of the boundary layer. Finally, these local coefficients are averaged to determine the overall convection coefficient. This method enables accurate modeling of heat transfer behavior in systems where the flow state evolves from laminar to turbulent, providing critical insights for engineering applications involving fluid dynamics and thermal management.

Taking water level 1 as an example, the cooling water flows through a 45 medium carbon steel substrate with a length of 0.14 m at a water velocity of 4.762 m/s. Assuming a steady state, the critical Reynolds number for transition is $R_{e_{x,c}}=5\times 10^{5}$. The local convection coefficients in the laminar and turbulent regions can be given as follows:

(4) $$h_{lam}\left(x\right)=C_{lam}x^{-0.5}$$

(5) $$h_{turb}\left(x\right)=C_{turb}x^{-0.2}$$

where, for a flat plate, the characteristic length is *x*, the distance from the leading edge.

When the water temperature is 283.15 K, Equations (6) and (7) can be given as follows:

(6) $$C_{lam,283.15}=367.366\;W/(m^{1.5}\cdot K)$$

(7) $$C_{turb,283.15}=1902.01\;W/(m^{1.8}\cdot K)$$

The constant C depends on the nature of the flow and the water temperature. The average water temperature was measured by a thermometer three times. The initial temperature of the water was 283.15 K.

When T = 283.15 K, $\;\rho =v_{f}^{-1}=1000\;kg/m^{3}$,$\;\;\mu =1308\times 10^{-6}\;N\cdot s/m^{2}$.

The local convection coefficient depends on whether there is laminar or turbulent state. Therefore, the existence range of these states must be determined the existence range of these states by solving the transition position *x_c_*.

In the calculation of the boundary layer, the representative critical Reynolds number is usually used as Equation (8):

(8) $${Re}_{x,c}\equiv \frac{\rho u_{\infty }x_{c}}{\mu }=5\times 10^{5}$$

Therefore, at 283.15 K,

(9) $$\bar{h}=\frac{1}{L}\int_{0}^{L}hdx=\frac{1}{L}\left[\int_{0}^{x_{c}}h_{lam}dx+\int_{x_{c}}^{L}h_{turb}dx\right]\;=\frac{1}{0.14\;m}\left[\frac{367.366\;W/\left(m^{1.5}\cdot K\right)}{0.5}\times \left(0.13734^{0.5}\right)m^{0.5}\;+\frac{1902.01\;W/\left(m^{1.8}\cdot K\right)}{0.8}\times \left(0.14^{0.8}-0.13734^{0.8}\right)m^{0.8}\right]\;=1998\;J/(m^{2}\cdot s\cdot K)$$

Similarly, the heat transfer coefficients corresponding to the water flow rates of 5.952 m/s and 6.25 m/s are $2360\;J/(m^{2}\cdot s\cdot K)$ and $2429\;J/(m^{2}\cdot s\cdot K)$, respectively.

## 3. Results and Discussion

### 3.1. Analysis of Nickel-Based Coatings with and Without the Water Cooling

**Figure 8:**
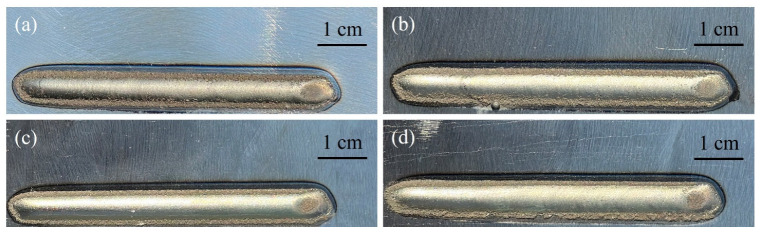
Macroscopic images of the coating surface at different water velocities: (**a**) 0 mL/s, (**b**) 200 mL/s, (**c**) 500 mL/s, and (**d**) 700 mL/s.

Compared with the traditional laser additive manufacturing process, the laser additive manufacturing process on the water-cooled substrate greatly improves the cooling rate of the coating and the substrate, which has an important influence on the macro forming, the dilution rate, and the surface roughness of the coating. Figure 8 demonstrates the macroscopic image of the coating corresponding to different process parameters. The surface of the coating is well formed. There are no obvious cracks, and the surface roughness has changed significantly. Figure 8a infers the Ni45 coating on the substrate without the water cooling. Comparing Figure 8a–c, it arises that with the increase in water velocity, the surfaces of the coatings are more smooth. Compared with Figure 8c, the surface roughness of the coating was increased in Figure 8d, which is due to the excessive cooling rate. The cooling rate is greatly increased during the deposition process due to the heat transfer effect of the water flow. As depicted in Figure 8d, the coating edge has an obvious tooth and irregular shape. The area values of unmolten powders at the end of the coating in Figure 8a–c were measured by ImageJ (1.54, National Institutes of Health, Bethesda, MD, USA) to be 0.155 cm^2^, 0.088 cm^2^, and 0.055 cm^2^, respectively. It can be found that the area value of the unmolten powder at the end of the coating layer decreases as the water velocity increases, while the corresponding area in Figure 8d is 0.074 cm^2^, which is due to the excessive cooling rate. This confirms that applying the water cooling to the substrate makes the laser energy more concentrated during the laser additive manufacturing process, which is beneficial to improving the surface quality of the coating.

### 3.2. Microstructure of Nickel-Based Coatings with and Without the Water Cooling

**Figure 9:**
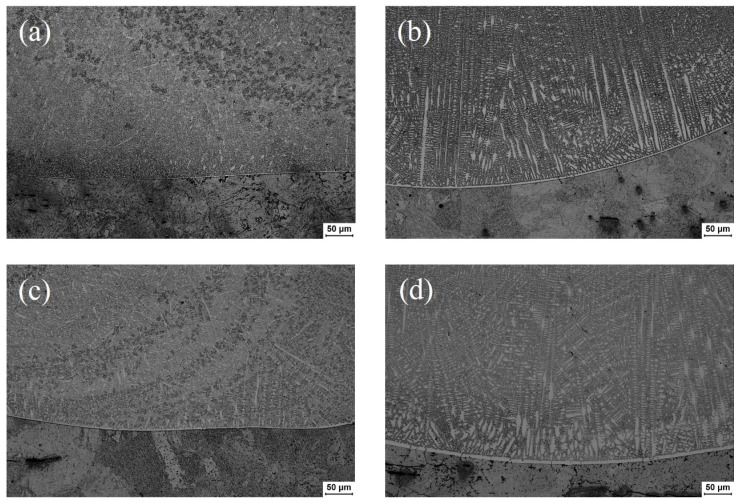
Microstructures at the bottom of the coating with different water velocities: (**a**) 0 mL/s, (**b**) 200 mL/s, (**c**) 500 mL/s, and (**d**) 700 mL/s.

**Figure 10:**
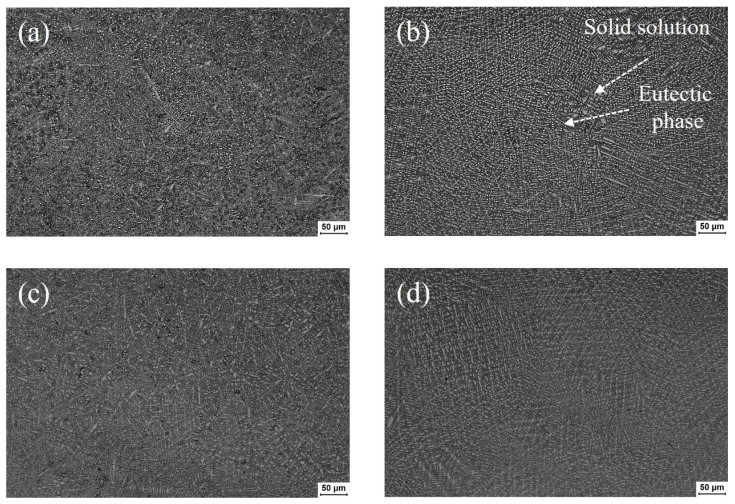
Microstructures at the middle of the coating with different water velocities: (**a**) 0 mL/s, (**b**) 200 mL/s, (**c**) 500 mL/s, and (**d**) 700 mL/s.

The microstructure of Ni45 coating is mainly composed of cellular, columnar, and equiaxed crystals. The coating is mainly composed of γ-Ni austenite grains with different morphologies, borides, silicides, and eutectics formed in interdendritic regions. The Ni45 alloy powder contains a high content of B and Cr elements. This makes the coating have a small amount of primary-precipitated chromium boride, which plays an important role in dispersion strengthening. Figure 9a and Figure 10a illustrate microstructure diagrams of the bottom and middle of the XOY section of the Ni45 alloy coating prepared by laser additive manufacturing without water cooling, respectively. Figure 9b–d and Figure 10b–d depict microstructures of the bottom and middle of the XOY section of the Ni45 alloy coating prepared by the laser additive manufacturing at different velocities of the water cooling.

As depicted in Figure 9, a large number of granular and short rod-like cellular crystals appear near the fusion line, and narrow dendrites appear along the growth direction of the cellular crystals. The results indicate that the dendrite morphology and the size of the coating are related to the cooling rate and the temperature gradient. The larger the cooling rate, the smaller the lamellar spacing of arborescent crystal, the finer the dendritic structure, and the higher the uniformity of the alloy composition and the phase distribution. After the application of the water cooling to the substrate, the degree of compositional segregation of the coating during the rapid cooling is reduced. In addition, the yield strength of the eutectic alloy is further improved with the increase in eutectic dispersion. Therefore, the refined structure can improve the hardness of the material.

Comparing Figure 9a,b, it can be found that after the application of the water cooling to the substrate, the cellular crystals at the bottom of the coating form columnar crystals that grow straight and regularly toward the center of the molten pool. Comparing Figure 9b,c, it can be concluded that with the increase in the velocity of the water cooling, the size of columnar crystals decreases. In the meanwhile, the number of and the length of secondary dendrites increase significantly. Figure 9b–d illustrate that with the further increase in water cooling velocity, the temperature gradient is larger. Moreover, the growth morphology at the end of small-area cellular crystals tends to form fine dendrites. Figure 9d states that the grain size of the columnar crystal becomes larger when the water velocity increases to 700 mL/s. This is due to the excessive cooling rate of the crystals caused by the excessive water velocity, which makes the growth time of the secondary dendrite to be too short.

Figure 10 expresses that the microstructure distributed in the middle of the coating is mainly the ‘cross-shaped’ dendritic crystals growing. According to Figure 10a,b, after the application of the water cooling to the substrate, the dendrite size at the top of the coating is significantly reduced, the number of long equiaxed crystals as well as ‘cross’ dendrites is significantly reduced, and it becomes a fine grain with dispersed distribution. At the same time, a large number of white solid solutions appeared in the microstructure. The area of white solid solutions in Figure 10a,b was measured by ImageJ software to be 13,153.8 μm^2^ and 18,710.4 μm^2^, respectively. It is obvious that the hardness of solid solutions like Cr_23_C_6_, Cr_7_C_3_, CrB, and other borides is generally higher than that of eutectic phases like carbides, γ-Ni austenite, borides, silicides, and γ-Ni austenite. Comparing Figure 10b,c, it can be concluded that the cooling rate of the coating increases, and the growth direction of the crystal is more messy when the water velocity increases and the gap between the broken dendrites increases. Furthermore, the gap between the broken dendrites increases, the finer grains are dispersed in the gap, and the number of fine white solid solutions increases significantly. Comparing Figure 10c,d, it can be concluded that the grain size is further reduced, the number of white solid solution phases is significantly reduced, and the growth direction of equiaxed crystals tends to be more regular with the further increase in water velocity.

**Figure 11:**
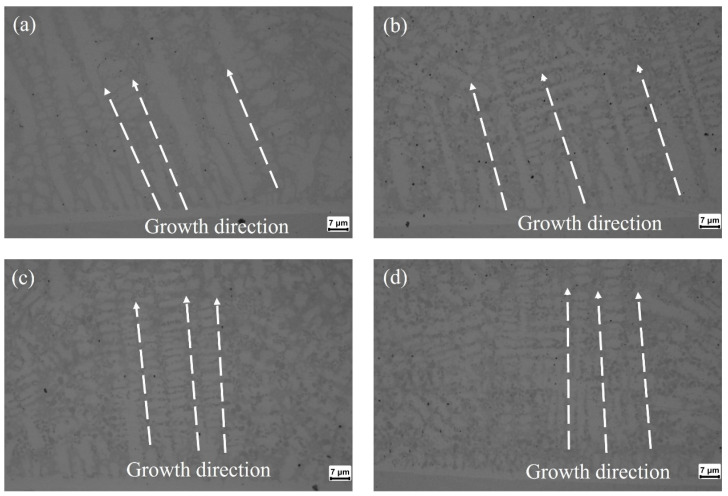
The YOZ cross-section microstructure of the coating at different water velocities: (**a**) 0 mL/s, (**b**) 200 mL/s, (**c**) 500 mL/s, and (**d**) 700 mL/s.

Figure 11 depicts the microstructure of the YOZ cross-section near the interface of coating/substrate at different water velocities. This figure indicates that the cooling rate of the coating gradually increases as the water flow rate increases. The angle between the dendrite growth direction and the fusion line near the weld junction was measured, and the average value was measured three times. The angles at no water cooling condition are 66.834°, while those at the water velocity of 200 mL/s, 500 mL/s, and 700 mL/s are 72.826°, 85.453°, and 87.295°. This means the angle between the dendrite growth direction and the fusion line increases with the increasing water velocity. In Figure 11a,b, it can be observed that the grain size of columnar crystals grown from the fusion line to the center of the coating and secondary dendrites with lateral growth appear when the water cooling is significantly reduced.

### 3.3. Hardness Analysis of Nickel-Based Coatings with and Without Water Cooling

The HXS-1000TAC microhardness tester (Shanghai Haowei Photoelectric Technology Co., Ltd., Shanghai, China) was used to test the hardness of the coating cross-section, with a load of 200 g and a load-holding duration of 15 s. The hardness values were measured at five hardness points in each zone at the same depth of the horizontal position and averaged to reduce the data error.

**Figure 12:**
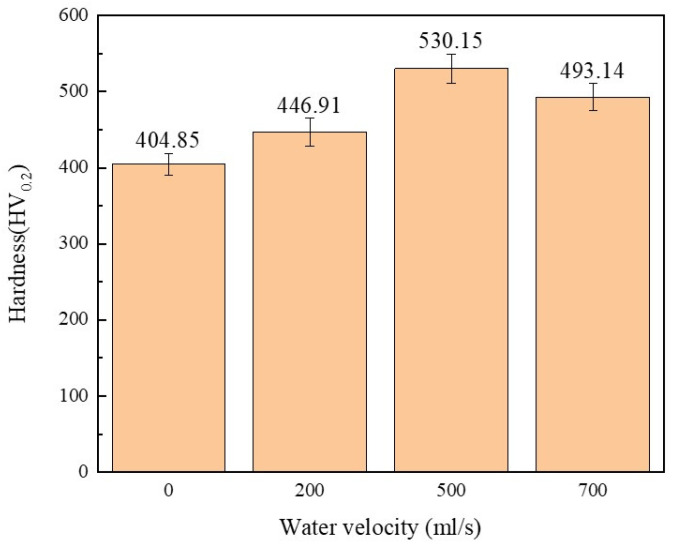
Average hardness of the coating at different water velocities.

Figure 12 shows the average hardness of the coating at different water velocities. This figure represents that the hardness of the coating obtained by the substrate with water cooling conditions of 200 mL/s and 500 mL/s is higher than the coating obtained by the substrate without water cooling. This is firstly due to the decrease in the dilution rate of the matrix under the water cooling condition, which enhances the solid solution strengthening effect of the alloy elements. Secondly, the water-cooled substrate significantly refined the grain size of the substrate and the coatings, resulting in fine-grain strengthening. According to Yang et al. [33] research, the coating application of the water cooling with a velocity of 700 mL/s to the substrate results in fewer elements of the substrate melting into the coating. Most of the elements of the coating do not dissolve to form carbides and borides such as Cr_23_C_6_, Cr_7_C_3_, and CrB in the coating to improve the hardness. Moreover, the number of compounds such as NiSi and NiB as hard compounds decreases in the coating, thereby reducing the hardness.

**Figure 13:**
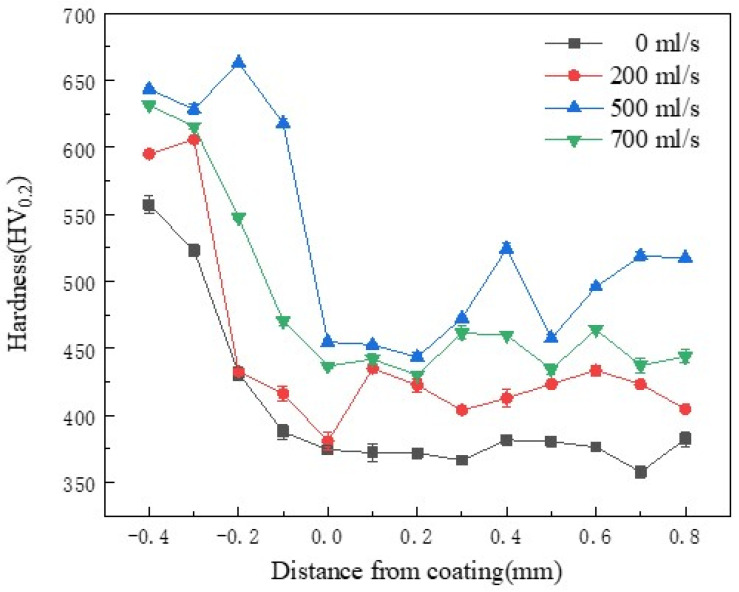
The hardness profile of the coating at different water velocities.

Figure 13 reflects the hardness distribution of the coatings subjected to the water cooling at different velocities. This figure infers that the hardness of the substrate near the interface of the coating/substrate is significantly higher than that of the coating. This is due to the quenching influence of the substrate during the laser cladding process. In addition, due to the application of the water cooling at the bottom of the substrate, the quenching effect is more significant. Compared with the heat-affected zone of the coating without water cooling condition, the average hardness is increased from 474.88 HV_0.2_ to 638.16 HV_0.2_, and the hardness is increased by about 34.3%.

### 3.4. Simulation Results and Analysis

**Figure 14:**
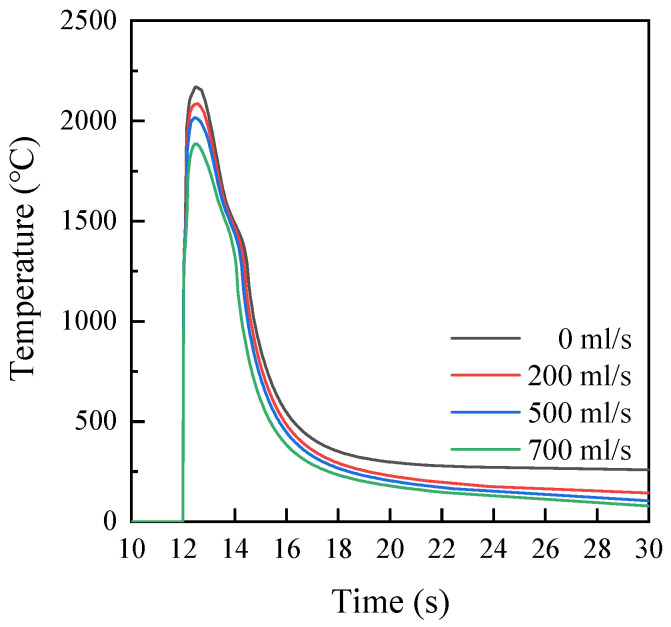
Thermal cycle curves of coating point x at different water velocities.

Figure 14 is the thermal cycle curve of the coating point x at different velocities of the water cooling. This figure depicts that the temperature of the point x begins to rise when the time reaches 12 s, and the temperature of the point x reaches the peak value at 12.5 s. The thermal cycle curve from 13.5 s to 14.5 s shows an obvious ‘step‘ shape, which is due to the supercooling degree. It can be seen from this figure that the peak temperature of point x gradually decreases with the increase in water velocity. In the meantime, the cooling rate of point x increases with the increase in water velocity after 15 s. This is because the heat transfer influence of the coating is considered a significant issue with the increase in water velocity.

**Figure 15:**
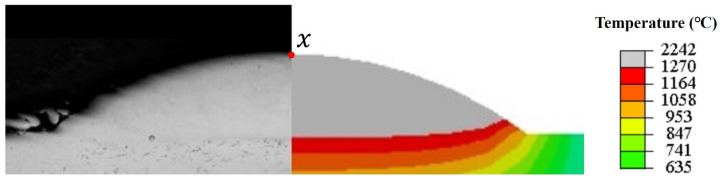
The temperature distribution of the laser additive manufactured coating at 12.5 s.

Figure 15 shows the temperature distribution of the coating without the water cooling. It can be seen from this figure that the numerical simulation results of the coating are in good agreement with the macroscopic cross-section in the experiment. The maximum temperatures of the molten pool center of the coating on the substrate without the water cooling and with different velocities of the water cooling are 2242 °C, 2173 °C, 2073 °C, and 1936 °C. The residence time above 1270 °C was 2.608 s, 2.472 s, 2.349 s, and 2.116 s. During the cooling stage, the cooling rate in the center of the molten pool gradually increased with the increase in water velocity. Because the flowing cooling water directly contacts the bottom of the substrate during the laser additive manufacturing process. Consequently, the heat exchange efficiency improves, and the solidification cooling rate of the molten pool accelerates.

In order to analyze the thermal behavior of each point in the process of laser additive manufacture, the temperature field of three points was selected from the model.

**Figure 16:**
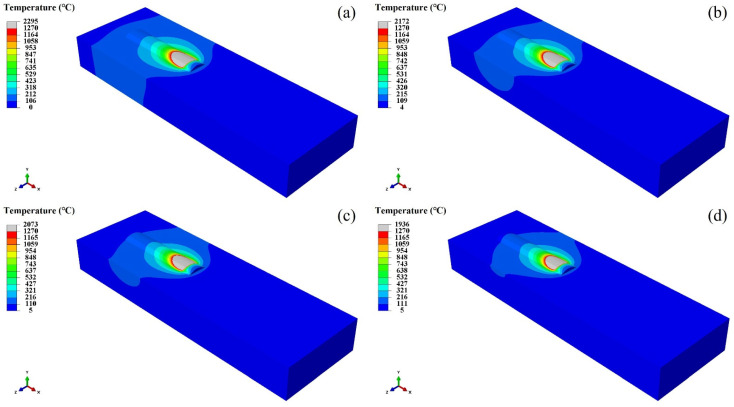
Temperature distribution of coating at 10 s of laser additive manufacturing under different water velocities: (**a**) 0 m/s, (**b**) 200 mL/s, (**c**) 200 mL/s, and (**d**) 700 mL/s.

Figure 16 expresses the temperature distribution of the coating when the laser additive manufacturing time is 14 s at different water flow rates. This figure indicates that the temperature of the coating molten pool has changed significantly with the increase in water velocity. Figure 6 also illustrates that the thermal conductivity of the material gradually decreases with the increase in temperature, which makes the heat dissipation condition of the produced part worse. Therefore, the temperature gradient around the molten pool decreases, the temperature of the molten pool increases, and the volume of the molten pool becomes larger with the increase in temperature. The heat dissipation efficiency increases with the increase in water velocity. This means that the temperature of the coating molten pool decreases with the increase in the water flow rate. At the same time, the temperature gradient around the coating molten pool increases with the increase in water velocity. Moreover, the volume of the molten pool under the water cooling condition is smaller than that under no water cooling condition.

**Figure 17:**
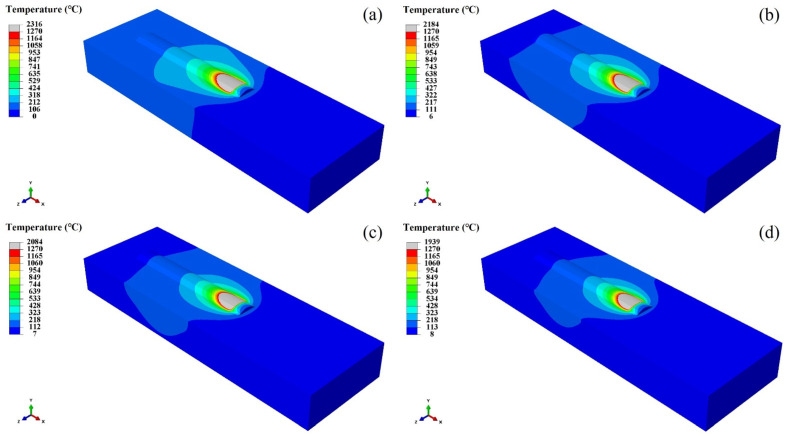
The temperature distribution of coating at 14 s under different water velocities: (**a**) 0 m/s, (**b**) 200 mL/s, (**c**) 200 mL/s, and (**d**) 700 mL/s.

**Figure 18:**
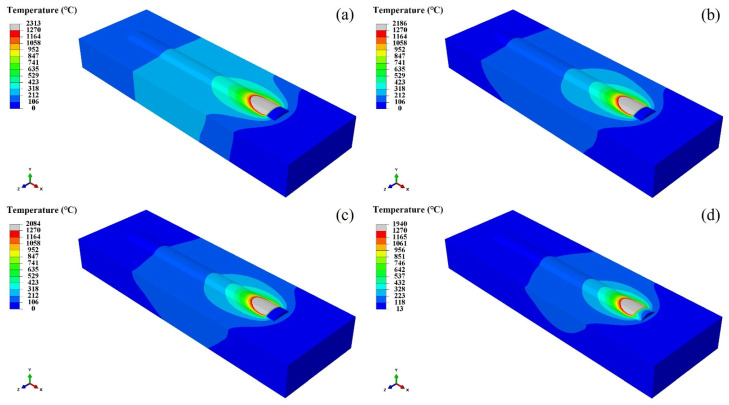
The temperature distribution of coating at 19 s under different water velocities: (**a**) 0 m/s, (**b**) 200 mL/s, (**c**) 200 mL/s, and (**d**) 700 mL/s.

Figure 17 and Figure 18 show the temperature distribution of the coating when the laser additive manufacturing at 14 s and 19 s, respectively, at different water velocities. These figures infer that the heat transfer effect of the water cooling is weakened with the increase in laser cladding time. The deposition time becomes longer, and the overall temperature of the substrate as well as the coating increases.

## 4. Conclusions

In this study, the laser additive manufactured Ni45 coating based on the water cooling substrate was studied by experimental and finite element simulation investigation. The main conclusions are as follows:

1. The hardness of 45 medium carbon steel substrate is about 200 HV_0.2_. The average hardness of Ni45 coating deposited by the laser additive manufacturing without water cooling is 404.85 HV_0.2_. After applying the water cooling and changing the water velocity, the average hardness of the coating was increased to 530.15 HV_0.2_, and the hardness was increased by about 31%;
2. The microstructure of Ni45 coating mainly consists of cellular crystal, columnar crystal, secondary dendrite, and equiaxed crystal. Compared with the Ni45 coating deposited by the laser additive manufacturing without the water cooling, the number of cellular crystals deposited by the laser additive manufacturing with different water velocities increased significantly, the grain sizes of columnar and equiaxed crystals were refined, and the number and length of secondary dendrites increased significantly. The above phenomenon becomes more obvious with the increase in water velocity;
3. The temperature field of the laser cladding Ni45 coating without the water cooling condition and the substrate with different water velocities was simulated by ABAQUS simulation software. It was found that the peak temperature of the center of the coating molten pool gradually decreased, and the cooling rate gradually increased with the increase in water velocity.

## Figures and Tables

**Table 1:** Chemical composition of Ni45 alloy powder.

C	Cr	Si	Mn	Fe	B	Ni
0.45	12.00	4.00	0.10	10.00	2.40	Bal

**Table 2:** Parameters of the laser additive manufacturing process.

Number	Laser Power	Powder Feed Rate	Scanning Speed	Gas Flow Rate	Water Velocity
	(W)	(g/min)	(mm/s)	(L/min)	(mL/s)
1	2600	12	3	10	0
2	2600	12	3	10	200
3	2600	12	3	10	500
4	2600	12	3	10	700

## Data Availability

The data presented in this study are available on request from the corresponding author due to the data that have been used are confidential.

## References

[1] Moeinfar K., Khodabakhshi F., Kashani-bozorg S.F., Mohammadi M., Gerlich A.P. (2022). A review on metallurgical aspects of laser additive manufacturing (LAM): Stainless steels, nickel superalloys, and titanium alloys. J. Mater. Res. Technol..

[2] Li T., Xu J., Bi X., Li R. (2022). Microstructure evolution and crack propagation mechanism during laser lap welding of Ti6Al4V and DP780 steel with CoCrNi powder. Mater. Des..

[3] Guo S., Xu D., Li Y., Guo Y., Wang S., Macdonald D.D. (2021). Corrosion characteristics and mechanisms of typical Ni-based corrosion-resistant alloys in sub- and supercritical water. J. Supercrit. Fluids.

[4] Zhu L., Yang Z., Xin B., Wang S., Meng G., Ning J., Xue P. (2021). Microstructure and mechanical properties of parts formed by ultrasonic vibration-assisted laser cladding of Inconel 718. Surf. Coat. Technol..

[5] Balaguru S., Gupta M. (2021). Hardfacing studies of Ni alloys: A critical review. J. Mater. Res. Technol..

[6] Hemmati I., Ocelík V., De Hosson J.T.M. (2013). Advances in Laser Surface Engineering: Tackling the Cracking Problem in Laser-Deposited Ni-Cr-B-Si-C Alloys. JOM.

[7] Ni M., Chen C., Xu R., Hosseini S.R., Li R., Zhang X., Zhou K. (2022). Microstructure and mechanical properties of additive manufactured Inconel 718 alloy strengthened by oxide dispersion with 0.3 wt% Sc addition. J. Alloys Compd..

[8] Chen Y., Lu F., Zhang K., Nie P., Hosseini S.R., Feng K., Li Z., Chu P.K. (2016). Investigation of dendritic growth and liquation cracking in laser melting deposited Inconel 718 at different laser input angles. Mater. Des..

[9] Armstrong M., Mehrabi H., Naveed N. (2022). An overview of modern metal additive manufacturing technology. J. Manuf. Process.

[10] Svetlizky D., Zheng B., Vyatskikh A., Das M., Bose S., Bandyopadhyay A., Schoenung J.M., Lavernia E.J., Eliaz N. (2022). Laser-based directed energy deposition (DED-LB) of advanced materials. Mater. Sci. Eng. A.

[11] Svetlizky D., Das M., Zheng B., Vyatskikh A.L., Bose S., Bandyopadhyay A., Schoenung J.M., Lavernia E.J., Eliaz N. (2021). Directed energy deposition (DED) additive manufacturing: Physical characteristics, defects, challenges and applications. Mater. Today.

[12] Dezaki M.L., Serjouei A., Zolfagharian A., Fotouhi M., Moradi M., Ariffin M.K.A., Bodaghi M. (2022). A review on additive/subtractive hybrid manufacturing of directed energy deposition (DED) process. Adv. Powder Mater..

[13] Lesyk D.A., Martinez S., Mordyuk B.N., Dzhemelinskyi V.V., Lamikiz A., Prokopenko G.I. (2019). Effects of laser heat treatment combined with ultrasonic impact treatment on the surface topography and hardness of carbon steel AISI 1045. Opt. Laser Technol..

[14] Chen C., Zeng X., Wang Q., Lian G., Huang X., Wang Y. (2020). Statistical modelling and optimization of microhardness transition through depth of laser surface hardened AISI 1045 carbon steel. Opt. Laser Technol..

[15] Kuai Z., Li Z., Liu B., Chen Y., Li H., Bai P. (2023). Microstructure and mechanical properties of CuCrZr/316L hybrid components manufactured using selective laser melting. J. Alloy Compd..

[16] Zhang J., Xue D., Cai X., Ding X., Ren X., Sun J. (2016). Dislocation induced strain glass in Ti_50_Ni_45_Fe_5_ alloy. Acta Mater..

[17] Sun S., Fu H., Ping X., Lin J., Lei Y., Wu W., Zhou J. (2018). Reinforcing behavior and microstructure evolution of NbC in laser cladded Ni45 coating. Appl. Surf. Sci..

[18] Wang Y., Wu G., Zhang L., Guo Y., Wang C., Li L., Xiong X. (2023). Microstructure evolution and mechanical properties of a cast and heat-treated Al–Li–Cu–Mg alloy: Effect of cooling rate during casting. Mater. Sci. Eng. A.

[19] Su Y., Hu B., Wang S., Yu X., Yang S., Wang S., Liu H. (2023). Effect of cooling rate after isothermal stage of vacuum austempering on microstructure and hardness of M50 bearing steel. J. Mater. Res. Technol..

[20] Han T., Chen J., Wei Z., Qu N., Liu Y., Yang D., Zhao S., Lai Z., Jiang M., Zhu J. (2023). Effect of cooling rate on microstructure and mechanical properties of AlCrFe_2_Ni_2_ medium entropy alloy fabricated by laser powder bed fusion. J. Mater. Res. Technol..

[21] Qin Q., Li J., Yang L., Liu L. (2024). Solidification behavior and microstructure of Ag–Cu eutectic alloy at different sub-rapid cooling rates. Mater. Chem. Phys..

[22] Gao L., Chuang A.C., Kenesei P., Ren Z., Balderson L., Sun T. (2024). An operando synchrotron study on the effect of wire melting state on solidification microstructures of Inconel 718 in wire-laser directed energy deposition. Int. J. Mach. Tools Manuf..

[23] Tamanna N., Crouch R., Naher S. (2019). Progress in numerical simulation of the laser cladding process. Opt. Laser Eng..

[24] Chen Y., Lu F., Zhang K., Nie P., Hosseini S.R.E., Feng K., Li Z. (2016). Dendritic microstructure and hot cracking of laser additive manufactured Inconel 718 under improved base cooling. J. Alloy Compd..

[25] Wu D., Liu H., Lu F., Ma G., Yan S., Niu F., Guo D. (2019). Al_2_O_3_-YAG eutectic ceramic prepared by laser additive manufacturing with water-cooled substrate. Ceram. Int..

[26] Nie J., Chen C., Liu L., Wang X., Zhao R., Shuai S., Wang J., Ren Z. (2021). Effect of substrate cooling on the epitaxial growth of Ni-based single-crystal superalloy fabricated by direct energy deposition. J. Mater. Sci. Technol..

[27] Chai Q., Zhang H., Fang C., Qiu X., Xing Y. (2023). Numerical and experimental investigation into temperature field and profile of Stellite6 formed by ultrasonic vibration-assisted laser cladding. J. Manuf. Process.

[28] Chen W., Xu L., Han Y., Zhao L., Jing H. (2021). Control of residual stress in metal additive manufacturing by low-temperature solid-state phase transformation: An experimental and numerical study. Addit. Manuf..

[29] Liu Y., Xu T., Zhang D., Yang W., Chen G. (2022). Numerical simulation and microstructure formation mechanism of Ni-based coating fabricated by laser on copper plate. Optik.

[30] Unni A.K., Vasudevan M. (2021). Determination of heat source model for simulating full penetration laser welding of 316 LN stainless steel by computational fluid dynamics. Mater. Today: Proc..

[31] Manca O., Morrone B., Naso V. (1995). Quasi-steady-state three-dimensional temperature distribution induced by a moving circular Gaussian heat source in a finite depth solid. Int. J. Heat Mass Transf..

[32] Hampali C.D. (2022). Numerical analysis of laminar to turbulent transition boundary layer flow. Mater. Today Proc..

[33] Yang S., Chen N., Liu W., Zhong M., Wang Z., Kokawa H. (2004). Fabrication of nickel composite coatings reinforced with TiC particles by laser cladding. Surf. Coat. Technol..

